# High Resolution Structure of the Mature Capsid of *Ralstonia solanacearum* Bacteriophage ϕRSA1 by Cryo-Electron Microscopy

**DOI:** 10.3390/ijms222011053

**Published:** 2021-10-13

**Authors:** Grégory Effantin, Akiko Fujiwara, Takeru Kawasaki, Takashi Yamada, Guy Schoehn

**Affiliations:** 1 CEA, CNRS, IBS, Université Grenoble Alpes, F-38000 Grenoble, France; guy.schoehn@ibs.fr; 2 Center for Food Science and Wellness, Gunma University 4-2, Aramaki, Maebashi, Gunma 371-8510, Japan; akiko_fujiwara@gunma-u.ac.jp; 3 Unit of Biotechnology, Division of Biological and Life Sciences, Graduate School of Integrated Science for Life, Hiroshima University, Higashi-Hiroshima 739-8530, Japan; takeru@hiroshima-u.ac.jp; 4 Hiroshima Study Center, The Open University of Japan, Hiroshima 730-0053, Japan; tayamad@hiroshima-u.ac.jp

**Keywords:** bacteriophage, structure, capsid, near atomic, electron microscopy

## Abstract

The ϕRSA1 bacteriophage has been isolated from *Ralstonia solanacearum*, a gram negative bacteria having a significant economic impact on many important crops. We solved the three-dimensional structure of the ϕRSA1 mature capsid to 3.9 Å resolution by cryo-electron microscopy. The capsid shell, that contains the 39 kbp of dsDNA genome, has an icosahedral symmetry characterized by an unusual triangulation number of T = 7, *dextro*. The ϕRSA1 capsid is composed solely of the polymerization of the major capsid protein, gp8, which exhibits the typical “Johnson” fold first characterized in *E. coli* bacteriophage HK97. As opposed to the latter, the ϕRSA1 mature capsid is not stabilized by covalent crosslinking between its subunits, nor by the addition of a decoration protein. We further describe the molecular interactions occurring between the subunits of the ϕRSA1 capsid and their relationships with the other known bacteriophages.

## 1. Introduction

Double stranded (ds) DNA bacteriophages of the *Caudovirales* order are composed of an icosahedral capsid attached to either a short tail (*Podoviridae*), a long flexible tail (*Siphoviridae*) or a rigid contracting tail (*Myoviridae*) [1,2]. Phage’s capsids are primarily composed of the major capsid protein (MCP), which assembles as hexamers and pentamers called the capsomers that ultimately form a closed icosahedral shell with the portal/connector complex located at one of the capsid’s vertices. Once the viral genome has been packaged in the capsid, the tail, which is assembled through an independent pathway, attaches to the portal end [3]. All known MCPs from the *Caudovirales* bacteriophages adopt a similar fold, first characterized in the *E. coli* bacteriophage HK97 [4]. The same fold was also later found in some capsids of archaeal and eukaryotic viruses [5]. Although the “core” building block of a bacteriophage’s capsid is very well conserved, each one has evolved independently and developed its own specificities, such that each capsid’s assembly is almost unique, and this results in capsids having a variety of shapes (isometric or prolate), sizes (~50 nm in diameter for T = 7 geometry to more than 150 nm for the largest “jumbo” phages) and complexities. In addition to the connector/portal complex, the simplest capsids are composed solely of MCP’s capsomers [6,7,8], while others (usually larger ones) are made of a much more diverse and complex set of proteins [9,10,11,12,13,14]. Another remarkable feature of bacteriophage’s capsids is that the network of proteins forming it has to sustain the high internal pressure (20–60 ATM) resulting from the packaged dsDNA [15,16,17]. Therefore, the bacteriophages have developed additional stabilization mechanisms to strengthen their capsid. In HK97, at each 3-fold axis, an auto catalyzed covalent bond between two MCPs of two different capsomers is formed that create interlaced loops (the chainmail mechanism) that prevents the capsid disassembly [18]. Other phages have one (or more) additional minor capsid protein(s) that binds to the MCPs towards the end of the capsid assembly pathway. These decoration proteins come in different flavors and differ in their oligomeric states and in their binding locations [9,10]. Interestingly, some bacteriophages, such as *E. coli* Lambda [19], *S. Typhimurium* Gifsy-2 [20], *R. solanacearum* ϕRSL1 [11], ϕRSL2 [13], *P. phenolica* ϕTW1 [21] and *Salmonella* Typhi YSD1 [22], have an additional trimeric protein at each 3-fold axis, which acts as a plug to cement the capsid structure and can be considered as an alternative to the chainmail mechanism of HK97, at least conceptually. At the same time, some capsids, including some from jumbo phages, are only built by the MCPs without any detected covalent crosslinking or any additional protein (P22 [8], T7 [7], SF6 [23] and ϕXacN1 [24] for example). In addition to these cement proteins, the presence of other so-called decoration proteins has been demonstrated. These are located at the outside of the capsid and are often made of immunoglobulin domain. The accepted hypothesis is that they serve as anchors to attach to different surfaces, allowing the phage to be transported before reaching its host [25].

In order to better understand bacteriophage structures and assemblies, their specificities and similarities, we focus our effort on the bacteriophages infecting *Ralstonia solanacearum* [11,13]. *R. solanacearum* is a gram negative bacteria infecting a wide range of plant hosts and is causing bacterial wilt in many different crops [26]. As the first complete genome of a *R. solanacearum* strain became available, the role of phages in the evolution of the various strains has been studied [27]. Several bacteriophages infecting a wide range of *R. solanacearum* strains have been identified, including three dsDNA *Myoviridae,* ϕRSL1 [28], ϕRSL2 [29] and ϕRSA1 [30], the first two being jumbo phages. The 39 kbp genome of ϕRSA1 has been sequenced and 51 potential open reading frames (ORF) have been assigned [30]. In this study, the ϕRSA1 bacteriophage has been purified from infected *R. solanacearum* and has been imaged at high resolution by cryo-electron microscopy (cryo-EM). Its capsid has been reconstructed by image analysis to 3.9 Å. The high resolution of the three-dimensional (3D) structure obtained allows the identification of the constituents of the capsid as well as the definition of the molecular contacts between them and their comparison with other known bacteriophages.

## 2. Results and Discussion

### 2.1. Structure of the ϕRSA1 Capsid by Cryo-EM

**Figure 1:**
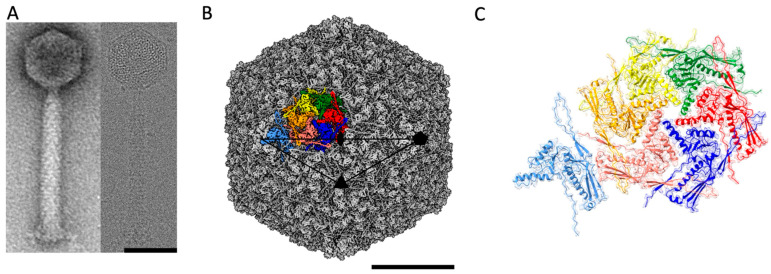
Cryo-EM structure of the ϕRSA1 capsid. (**A**) View of a ϕRSA1 mature phage imaged by negative stain (left panel) and cryo-EM (right panel). (**B**) Isosurface representation of the 3D reconstruction of the mature ϕRSA1 capsid at 3.9 Å resolution. The position of the symmetry axis (2, 3 and 5-fold) of the icosahedral capsid are indicated. The black triangle delimits the asymmetric unit of the capsid. The seven quasi equivalent subunits of the capsid asymmetric unit are highlighted in different colors. (**C**) Zoomed view on the same asymmetric unit with the seven quasi equivalent subunits of the major capsid protein represented as ribbons fitted in their corresponding Coulomb potential density (in light transparent shade). The scale bars are 50 and 20 nm in (**A**) and (**B**) respectively.

ϕRSA1 is composed of an isometric capsid (maximum diameter of 640 Å) attached to a long contractile tail of ~1500 Å (Figure 1A) which is a landmark of the *Myoviridae* subfamily of the dsDNA bacteriophages. The ratio between the lengths of the ϕRSA1 tail and capsid (2.3 for ϕRSA1) is quite high compared to other *Myoviridae* (0.8, 0.9 and 1.2 for T4, ϕRSL1 and ϕKZ respectively). This ratio for ϕRSA1 is closer to the one found in phages of the *Siphoviridae* family, which have a long, non-contractile tail (ratios of 2.9, 2.8 and 2.2, respectively, for phages HK97, T5 and SPP1 for instance). From cryo-EM images, the 3D structure of the ϕRSA1 capsid has been solved to an average resolution of 3.9 Å (Figure 1B and Figure S1). The capsid follows an icosahedral symmetry characterized by a triangulation number T = 7, *dextro* (d) in which the 11 out of the 12 vertices are made of pentamers (the last one is the portal connected to the tail) while the flatter facets are made of hexamers. At that resolution, we were able to build an atomic model of the major capsid protein gp8 (Table S1) which turned out to be the only constituent of the capsid’s pentamers and hexamers, even though gp10 could have been considered as a putative minor capsid protein as it has some sequence similarities to the head stabilization protein gpL of bacteriophage ϕRSc1935 [28]. It cannot be totally excluded that gp10 does not follow the icosahedral symmetry and is therefore not visible in our reconstruction. The attempts to resolve gp10 by either reducing the symmetry to C1 or by symmetry expansion of the capsid solved with icosahedral symmetry didn’t show any additional density which could be attributed to gp10. Accordingly, there are seven unique quasi equivalent subunits of gp8 that composed the asymmetric unit of the ϕRSA1 capsid (Figure 1C) for a total of 415 subunits in the capsid. The T = 7, d is quite unusual within the known phage’s symmetries and has only been described twice for the *Lactococcal* phage 1358 capsid [31] and the *E. Coli* P2 procapsid [32] while its mirrored geometry, T = 7, *laevo* (l), is much more common [33]. Sixteen different capsids of dsDNA phages are having a T = 7, l geometry in the Viper database (viperdb.scripps.edu/TNumber_Index.php) [4,22,23,34,35,36]. There is currently no real explanation for this discrepancy as for both T = 7, l and d, the network of interactions between MCPs are very similar (see below).

### 2.2. Structure of the ϕRSA1’s MCP

**Figure 2:**
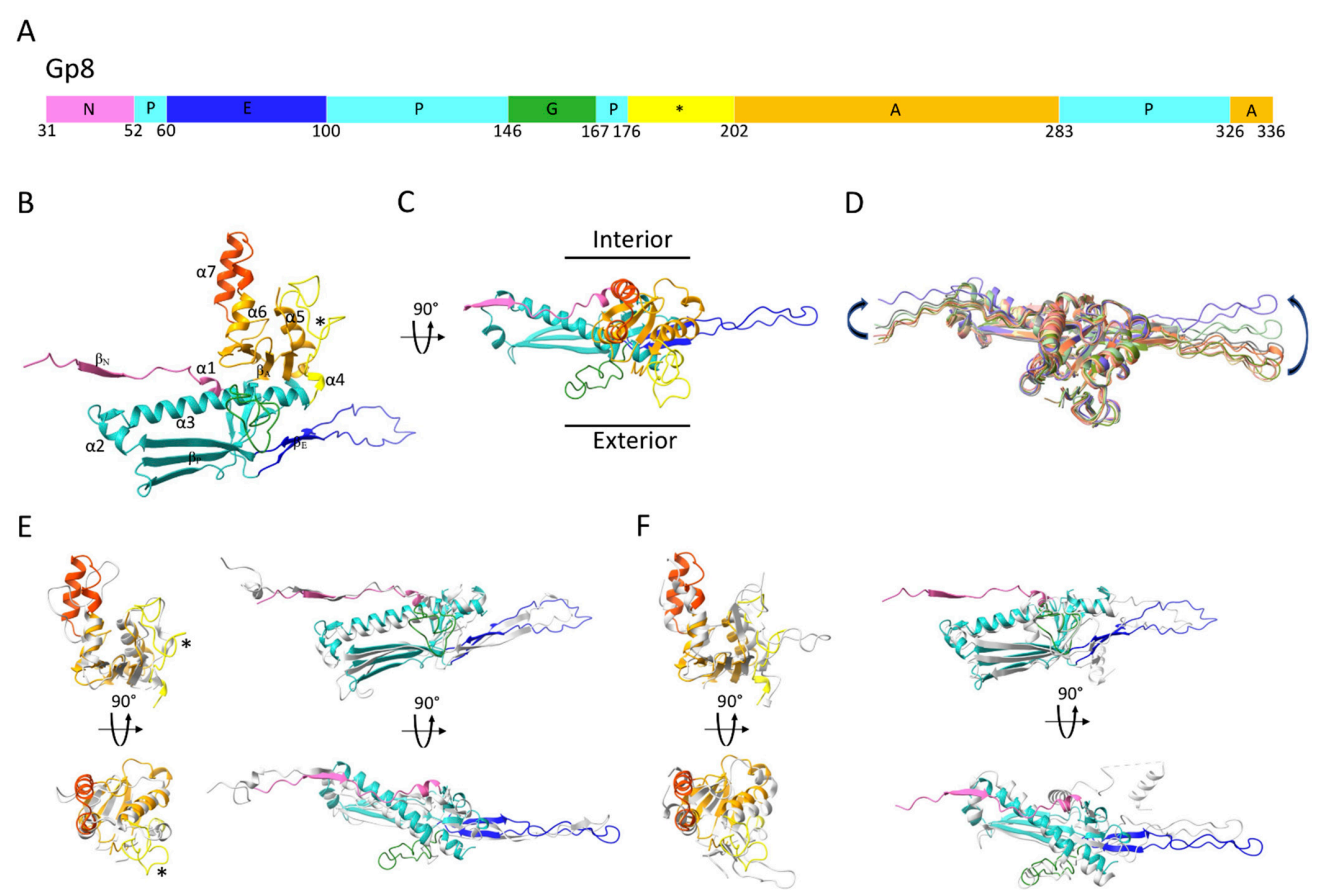
(**A**) Domain organization of ϕRSA1’s MCP, gp8. The main structural domains are indicated and colored. (**B**,**C**) Ribbon representation of one ϕRSA1 major capsid protein color coded according to the schematic in (**A**). The two major differences in conformation between the A domains of the ϕRSA1 and HK97 MCPs are further colored dark orange and yellow (indicated with a *). (**D**)- Superimposition of the seven pseudo-equivalent MCPs (aligned through their A and P domains) composing the asymmetric unit of the ϕRSA1 capsid. The two arrows indicate the amplitude of the conformational changes between the N domains and the E-loops of the different subunits. (**E**,**F**) Structural alignment of the ϕRSA1 MCP (colored ribbon) with the HK97 (**E**) and P4 (**F**) (grey ribbon) MCPs calculated with DALI. For both panels and for clarity, the superimpositions have been split between the A domains (left column) and the N, P and E domains (right column). Each superimposition is shown in two different views related by 90°.

The ϕRSA1’s MCP has the same characteristic fold which was first described for HK97’s MCP [4] and later found in all *Caudovirales* dsDNA phages, as well as in some *Archeal* viruses [5] and in the *Herpesviridae* family [37]. The core of the subunit is composed of the Axial (A) and Peripheral (P) domains to which the more flexible N-terminal (N) domain and Extended (E) loop are attached (Figure 2A–C). Whereas the ϕRSA1’s capsid MCP core remains very similar (mean RMSD = 0.94 ± 0.16 Å) between the seven pairs of MCPs of the asymmetric unit, the N domain and the E loop can be displaced by as much as 12 and 20 Å, respectively, between the seven quasi equivalent subunits of the asymmetric unit (Figure 2D) in order to adapt to different local environments. These displacements are in the same order of magnitude as what has been observed for other phages, irrespective of their dimensions (i.e., with smaller or larger T number) and are necessary to accommodate the small variations in local environment between the seven quasi equivalent subunits.

The A domain of the ϕRSA1 MCP is composed of a four stranded β sheet (β_A_) and four α helices (α4 to 7) (Figure 2B,C). It differs from HK97’s A domain in two regions (Figure 2E): 1- Residues 239 to 261 in ϕRSA1 form two α helices (α6 and 7) near the center of the capsomer while the corresponding region in HK97 is composed of loops (Figure 2E, orange color). 2- Residues 176 to 202 in ϕRSA1 form a longer loop than in HK97 which protrudes from the capsid’s surface (Figure 2E, yellow color). The A domains are involved in interactions between subunits of the same capsomer (intra capsomer interactions, see below).

The ϕRSA1’s P domain is composed of two consecutive α helices (α2 and 3) and a three stranded β sheet (β_P_). The longest “spine” helix (α3) is interrupted near its end by a long (22 residues in length) G-loop (Figure 2A–C). Such long insertion in the spine helix has been previously described in other bacteriophages [21], as well as in some archeal viruses [38,39] where it was shown that the longer G-loop was important to lock the E-loop in position. The fold of the ϕRSA1’s MCP is strongly similar to the one of the satellite bacteriophage P4 of *E. coli* (PDB 7JW1 [40], 56% identity of sequence) (Figure 2F). The structural similarities are striking for the A and P domains (including the G-loop), while the N domain and E loop are completely different as the structure of the P4’s MCP is from a procapsid form, a different assembly intermediate than the mature capsid’s form of ϕRSA1. And it has been shown for several bacteriophages that the major differences between the procapsid and the mature MCP’s folds are located in the N domain and the E-loop. Indeed, during the procapsid to mature capsid transition, the capsid shell is switching from a more spherical form to a more angular one which require extensive rearrangements [41].

### 2.3. Intra-Capsomeric Molecular Interactions between the Capsid’s MCPs

**Figure 3:**
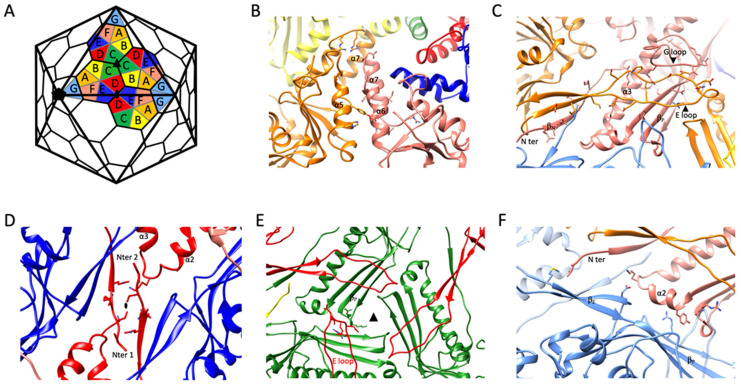
(**A**) Schematic view of the hexamers and pentamers arrangement in a T = 7, d capsid. The seven subunits (named A to G) that constitutes the asymmetric unit are highlighted. (**B**–**F**) Zoomed views on the five main interacting sites that exist between the MCPs of the ϕRSA1 capsid. For each panel, the color of the subunits represented is related to the color code shown in the schematic in (**A**). The side chains of the residues forming putative hydrogen bonds are also shown. (**B**) Interactions between two A domains of two MCPs (subunits A (orange) and F (salmon)) of the same capsomer. (**C**) Interactions between the P- and N- domains of a given subunit (subunit F—salmon) and the E-loop of a neighbor subunit of the same capsomer (subunit A—orange). (**D**) Interaction occurring at the 2-fold axis between two N-termini from two MCPs of two different capsomers (subunits D—red). (**E**) Interaction occurring at the 3-fold axis between the E-loop of a given subunit (subunit D—red) and the P-domain of a MCP of a neighbor capsomer (subunit C—green). (**F**) Interaction taking place between the P-domain of a subunit (subunit F—salmon) with the P- and N- domains of a MCP from a different capsomer (subunit G—light blue).

In total, each MCP interacts with eight others: two are from the same capsomer, while the other six are spread across three different adjacent capsomers. The analysis of the various subunit–subunit interactions reveals that there are five main interfaces which can explain all the intra and inter capsomer’s interactions (Figure 3).

Each MCP that belongs to a given capsomer (pentamer or hexamer) interacts with two of its immediate neighbors. The first main interaction site is between two A domains of two successive subunits. Helix α6 of a given subunit lines up with α5 from a subunit located counterclockwise from the reference subunit while two α7 helices of the same subunit pair pack head to tail near the center of the capsomer to complete this interface (Figure 3B).

The second main intra capsomeric interaction occurs between the N and P domains of a subunit with the E loop of a neighbor subunit (still counting counterclockwise). The latter extends over the N and P domains (including the long spine helix α3 and the three stranded β sheet (β_P_)). Near its tip, the E loop is further stabilized by the G loop of the reference subunit (Figure 3C). The G-loop of ϕRSA1, which is longer than in most phages, acts as a sort of lock for the E-loop. This interaction, repeated five or six times per capsomer, contribute significantly to the sturdiness of the capsid. In that regard, the G-loop of ϕRSA1 resembles the ones described in thermophilic viruses P74-26 and P23-45 where the longer G-loop has been shown to be one of the main determinants leading to the formation of an un-usually large T = 7 capsid [38,39].

### 2.4. Inter-Capsomeric Molecular Interactions between the Capsid’s MCPs

Each ϕRSA1’s MCP interacts with other MCPs of three different capsomers through three distinct interfaces. The first and second ones are located around each 2-fold (Figure 3D) and 3-fold (Figure 3E) axis, respectively, while the third one is between the P domains of two subunits (Figure 3F). The former occurs between two near perpendicular surfaces formed on one side by the three stranded and two stranded β sheets of the P domain (β_P_) and of the E loop (β_E_), respectively, and on the other side by the most distal regions of the P and N domains of the other subunit (Figure 3F).

The interface at the 2-fold axis involves mainly the terminal region (residues 31 to 37) of two N domains of two subunits belonging to two different capsomers. The interaction in the area is further strengthened by the close proximity of two P domain’s loops linking α2 to α3 (Figure 3F).

Finally, at each 3-fold axis, the tip of the E loop (residues 79 to 85) of a given subunit packs along the small loops (residues 110 to 112 and 304 to 307), which are parts of the three stranded β sheet of the P domain (β_P_) of another subunit from a different capsomer (Figure 3E). The 3-fold axis is often the place where specific stabilization mechanisms have been described for other phages. One such mechanism involves the binding of an additional minor capsid protein [11,20,21,42] and a second one is characterized by the formation of a covalent bond between the two subunits interacting at each 3-fold axis to form a chainmail like assembly at the capsid surface [18]. Interestingly, ϕRSA1 doesn’t display either additional densities or any continuous density between the two subunits interacting at the 3-fold axis, which leads to the conclusion that ϕRSA1 uses none of the most common mechanisms to stabilize its capsid at the 3-fold. It follows that ϕRSA1 solely relies on interactions between its MCPs to get a strong enough assembly which can sustain the high pressure inside the capsid caused by the packaging of its genome.

### 2.5. Comparison between the Right-(Dextro) and Left-(Laevo) Handed T = 7 Lattices

From the geometric principles of constructing an icosahedral lattice made of hexamers and pentamers having a defined T number, it is known that for some odd T numbers, two enantiomeric lattices can be build [43]. It is the case for T = 7 where both laevo and dextro hands are possible. The MCP fold of ϕRSA1 is similar to its equivalent in HK97, a well-studied T = 7, l capsid (Figure 2). The similarities also extend to the interaction sites within the capsomers and between the capsomers (Figure S2). In particular, the motifs and domains that were shown to be important in ϕRSA1 for the interaction at the 2-fold (the N domains) and at the 3-fold axis (the E-loop and P domain from two subunits of two different capsomers) are conserved in HK97 (Figure S2) and other T = 7, l capsids such as SPP1 [35] (Figure S2). If a similar convention is used for the names of the seven subunits composing the asymmetric unit (named A to G) (Figure S2) then the main difference between a T = 7, l and d lattice is which subunits are involved in the interaction at the 2-fold axis (subunits D and C for ϕRSA1 and HK97 respectively) and at the 3-fold axis (subunits C and D for ϕRSA, D and E for HK97). These discrepancies result in a different orientation of the MCPs involved in the interaction. For instance, the two N domains interacting at the 2-fold axis are rotated by nearly 90° between ϕRSA1 (Figure 3D) and HK97, SPP1 (Figure S2). However, these topological differences don’t prevent that the same type of molecular interactions between subunits are retained for both icosahedral geometries. The same observation was made for the procapsid of P2 phage from *E. Coli* [32] which exhibit also a T = 7d capsid.

## 3. Conclusions

The ϕRSA1’s mature capsid structure was solved to high resolution by cryo-EM, and is characterized by a right-handed (*dextro*) icosahedral lattice (T = 7, d). The ϕRSA1’s capsomers that constitute the capsid are solely composed of the MCP gp8. Different MCP’s interfaces which mediate the protein–protein interactions in the capsid have been identified. Two of them are involved in intra capsomer contacts, while the other three are engaged in inter capsomer interactions. These five key interactions are enough to create a sturdy network of proteins which can efficiently protect the viral genome and also resist the huge amount of pressure inside the capsid due to the densely packed DNA. It appears that ϕRSA1 follows very similar assembly principles and molecular organization as the other known capsids of the *Caudovirales* order, including the ones which have left-handed (*laevo*) icosahedral lattices. Even though dsDNA phage capsids display very different sizes, symmetries and shapes, they all obey the same building principles, which are reflected by the well conserved fold of their MCPs. Therefore, ϕRSA1 is another example of the extraordinary diversity of capsid’s designs that exists among the *Caudovirales* order.

## 4. Materials and Methods

### 4.1. Bacteriophage Production and Purification

*Ralstonia* bacteriophage ϕRSA1 was isolated in Japan and characterized as described before [27]. It was propagated with *Ralstonia solanacearum* M4S as the host. Host bacterial cells were cultured in CPG medium containing 0.1% (w/v) casamino acids, 1% (w/v) peptone, and 0.5% (w/v) glucose at 28 °C with shaking at 200–300 rpm. When the cultures reached an OD_600_ of 0.1 units, the phage was added at a multiplicity of infection (MOI) of 0.01–0.05. After culturing for a further 16–18 h, the cells were removed by centrifugation in the R12A2 rotor of a Hitachi Himac CR21E centrifuge (Hitachi Koki Co. Ltd., Tokyo, Japan), at 8000× *g* for 15 min at 4 °C. To increase phage recovery, ethyleneglycoltetraacetic acid (EGTA; final concentration, 1 mM) was added to the phage-infected culture at 6 to 9 h post infection [30]. The supernatant was passed through a 0.2 µm membrane filter followed by precipitation of the phage particles by centrifugation in a RPR20-2 rotor of a Hitachi Himac CR21E centrifuge at 40,000× *g* for 30 min at 4 °C. The bacteriophage particles were dissolved in 50 mM Tris-HCl at pH 7.5, 100 mM NaCl, 10 mM MgSO_4_, and stored at 4 °C before use. For further purification, the phage suspension was layered on a step-wise gradient of CsCl (ρ = 1.45, 1.50, and 1.75) and centrifuged with a P28S rotor in a Hitachi Himac CP80WX ultracentrifuge at 87,000× *g* for 2 h at 15 °C. Collected phage bands were dialyzed against 50 mM Tris-HCl at pH 7.5 containing 10 mM MgCl_2._

### 4.2. Negative Staining

Four µL of the bacteriophage sample (~0.1 mg/mL) were injected at the mica-carbon interface as described [11]. The sample was stained using 2% ammonium molybdate pH 7.5 and air-dried. Images were taken under low-dose conditions with a T12 transmission electron microscope (FEI) working at 120 kV and with a nominal magnification of 30,000 using an Orius SC1000 CCD camera.

### 4.3. Cryo-Electron Microscopy and Preprocessing

Three µL of sample were applied to 1.2/1.3 Quantifoil holey carbon grid (Quantifoil Micro Tools GmbH, Jena, Germany) and the grids were plunged frozen in liquid ethane with a Vitrobot Mark IV (Thermo Fisher Scientific, Waltham, USA) (3 s blot time, blot force 0). The sample was observed with a Polara Tecnai F30 electron microscope (FEI, Eindhoven, the Netherlands) at 300 kV. In total, 1470 images were recorded on a K2 summit direct detector (Gatan Inc., Pleasanton, USA) at 31k (calibrated pixel size of 1.21 Å/pixel) with Latitude S. Movies were recorded for a total exposure time of 8 s and 200 ms per frame resulting in 40 frame’s movies with a total dose of ~40 e^−^/Å^2^. Movies were motion corrected with motioncor2 [44] and CTF parameters were determined for each micrograph with gctf [45].

### 4.4. Image Analysis of the Capsid

A preliminary medium resolution model of the capsid has been obtained with the model based PFT2/EM3DR2 package [46]. Around 500 capsids have been selected by hand into 660 × 660 pixels^2^ boxes using X3d [47] and corrected for the CTF. The 3D structure of the capsid of ϕRSL1 [11] has been scaled to the same size as ϕRSA1, low-pass filtered to 50 Å and used as starting model. The model was refined to 9 Å resolution.

Then, the capsids were automatically picked from all the micrographs with Gautomatch [45] and coordinates were imported in Relion 3.07 [48], all software being maintained by SBGrid [49]. Two rounds of 2D classification were first performed to remove bad particles. The resulting particles were then refined imposing icosahedral symmetry with 2x binned images followed by a 3D classification using a mask which excludes the viral DNA and by another 3D refinement with the same 3D mask. The particles were then re-extracted un-binned in a box size of 660 pixels in Relion 3.1. A first 3D refinement gave a 3D reconstruction at 4.6 Å resolution. A first CTF refinement round (magnification anisotropy, beam tilt, trefoil, 4th order aberration and per particle defocus and astigmatism) with 100 particles per optic group (to account for the variation in coma alignment over the data collection) followed by a 3D refinement leads to a 3D reconstruction at 4.4 Å resolution. Then, particle polishing followed by two rounds of CTF refinements improved the resolution to 3.9 Å as determined by Fourier Shell Correlation (FSC = 0.143). More CTF refinements or 3D classifications as well as an attempt to correct for the Ewald sphere curvature did not improve further the resolution. The final 3.9 Å map was calculated from 3399 particles.

### 4.5. Model Building of the ϕRSA1 Capsid

An initial model for the ϕRSA1 MCP monomer was obtained with the I-Tasser web server [50] which was then rigid body fitted in the cryo-EM map with Chimera [51]. Then, the model was improved by several iterations of manual re-buildings in Coot [52] and of refinements in Phenix [53] and Rosetta [54]. This monomer model was then duplicated seven times to generate a first model of the complete asymmetric unit of the capsid. Then, the asymmetric unit’s model was improved by several iterations of manual re-buildings in Coot (mostly the N domains and the E-loops) and of refinements in Phenix and Rosetta. Finally, in order to fix clashes between asymmetric units and to avoid refining a complete model of the capsid which was too computer intensive, the subunits surrounding one complete asymmetric unit were added and this model, representative of all the possible interactions between subunits of the ϕRSA1 capsid, was improved by few iterations of manual re-buildings in Coot and of refinements in Rosetta and Phenix.

## Data Availability

The ϕRSA1 capsid map has been deposited in the EMDB (entry 13120). The atomic model derived from the cryo-EM map has been deposited in the Protein Data Bank (PDBid: 7OZ4).

## Supplementary Materials

Supplementary materials are available online at www.mdpi.com/article/10.3390/ijms222011053/s1.

## Abbreviations

3D	Tri-dimensional
Cryo-EM	Cryo Electron Microscopy
DALI	Distance mAtrix aLIgnment
dsDNA	Double Stranded DeoxyriboNucleic Acid
MCP	Major Capsid Protein
RMSD	Root Mean Square Deviation
