# Supplementary material for: High Resolution Structure of the Mature Capsid of Ralstonia solanacearum Bacteriophage ϕRSA1 by Cryo-Electron Microscopy
Source: Int J Mol Sci. 2021 Oct 13;22(20):11053. doi: 10.3390/ijms222011053 (PMC8538268; doi:10.3390/ijms222011053)
Supplement: Supplementary file 1 [file ijms-22-11053-s001.zip › ijms-1395092-supplementary.pdf]

Figure S1

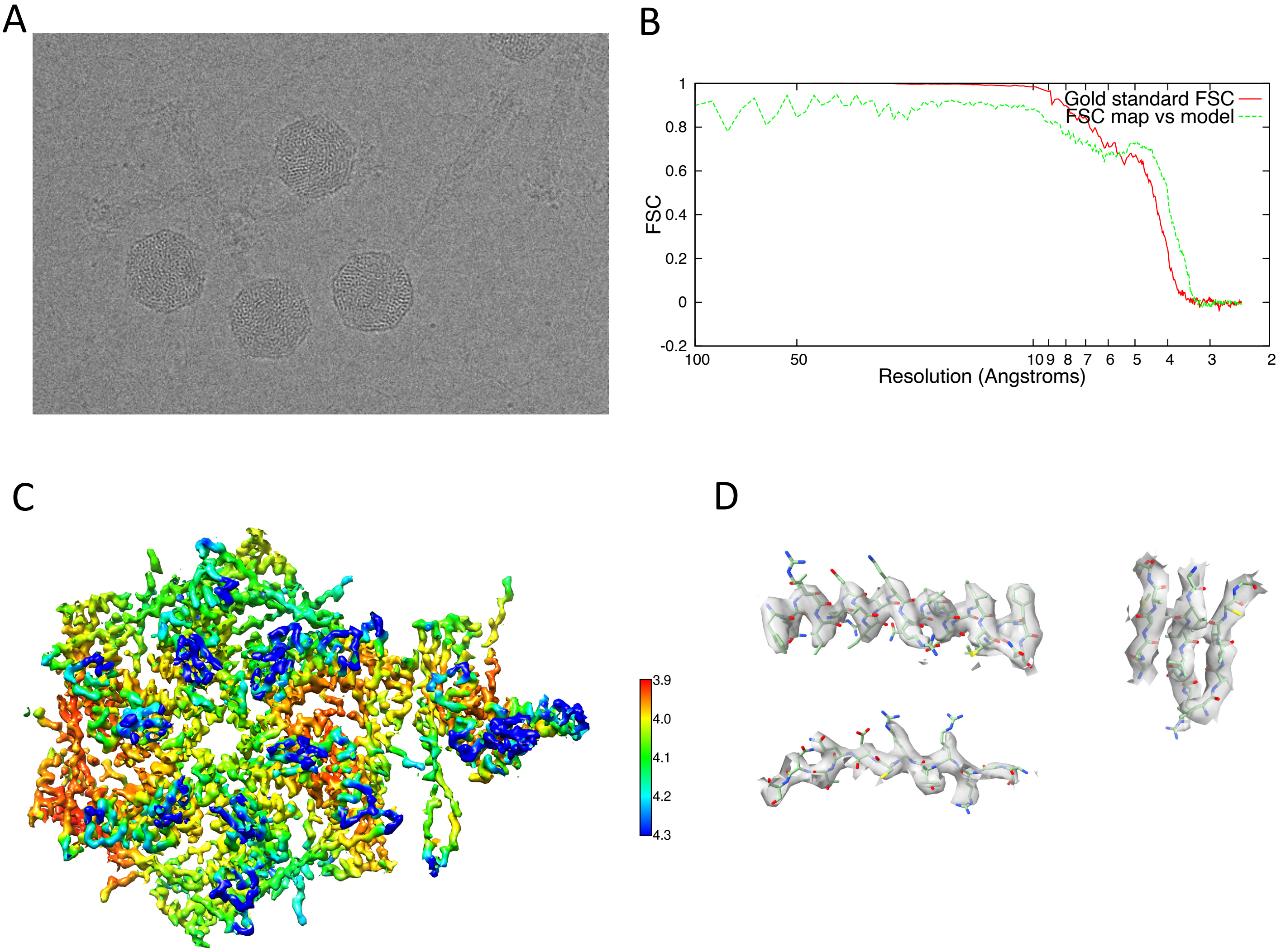

Figure S1. **Cryo-EM reconstruction of ϕRSA1 capsid** (**A**)- A cryo-EM field of view ϕRSA1 particles. (**B**)-Fourier Shell Correlation (FSC) curves calculated between two independent 3D reconstructions of the ϕRSA1 capsid (gold standard FSC) and between the cryo-EM 3D reconstruction and the build atomic model (FSC map vs model). (**C**)- Asymetric unit of the cryo-EM 3D reconstruction of ϕRSA1 capsid colored by local resolution. (**D**)- Three different areas of ϕRSA1's MCP are shown to illustrate both the high resolution of the 3D reconstruction (grey isosurface) and the quality of the atomic model built from it (represented as sticks).

Figure S2

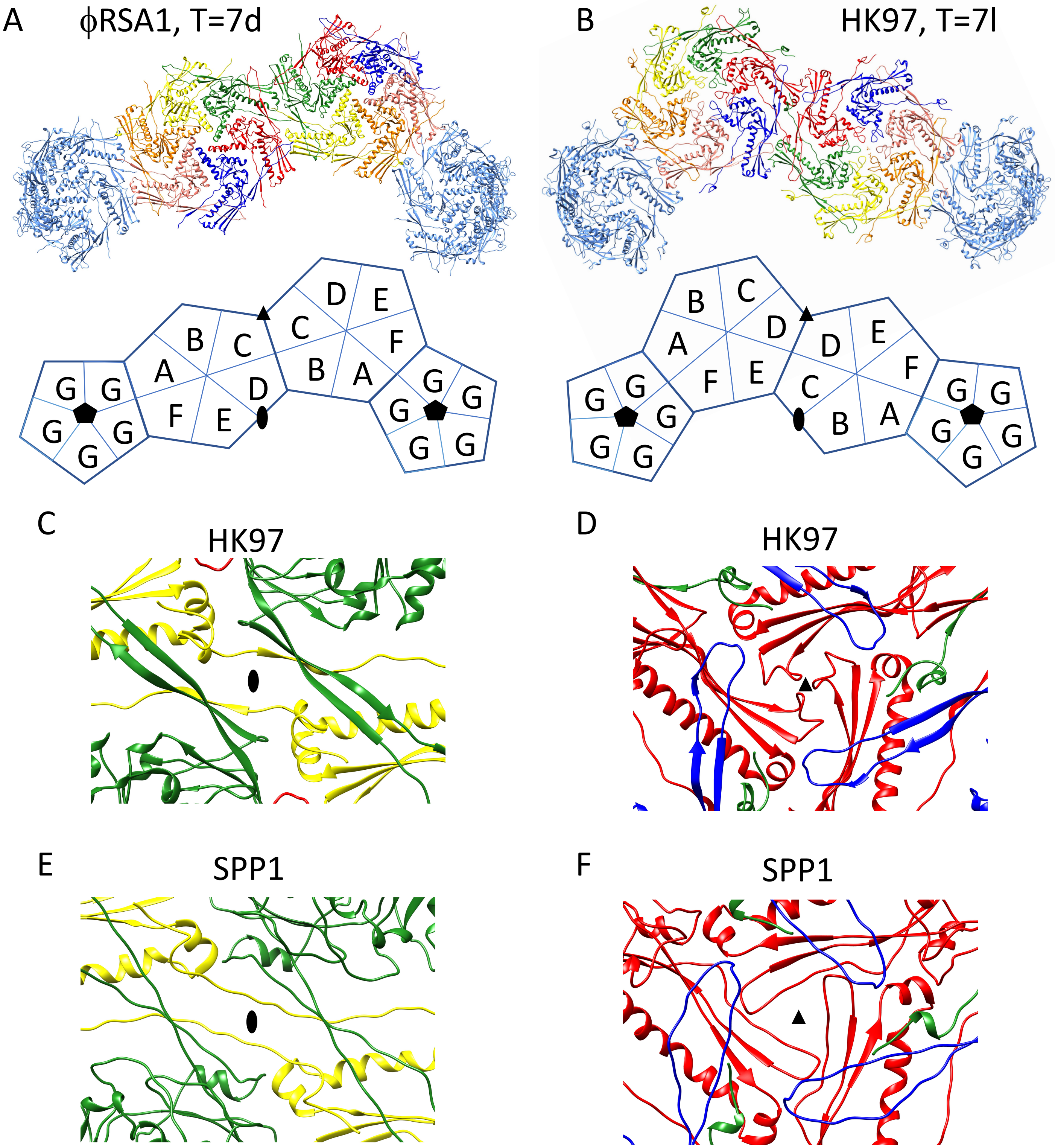

Figure S2: Comparison between T=7, l and T=7, d lattices A, B- View of the pentamers and hexamers lattices for ϕRSA1 (**A**) and HK97 (**B**) and their corresponding schematics . C & E- View of the interaction occurring at the 2-fold axis between two N-termini from two MCPs of two different capsomers (subunit B – yellow) of HK97 (**C**) and SPP1 (**E**) (for comparison with Figure 3D). D & F- View of the interaction occurring at the 3-fold axis between the E-loop of a given subunit (subunit E – blue) and the P-domain of a MCP of a neighbor capsomer (subunit D– red) for HK97 (**D**) and SPP1 (**F**) (for comparison with Figure 3E).

Table S1. Summary of data collection and atomic model statistics.

**Data collection**	
Microscope	Polara (FEI)
Voltage (kV)	300
Magnification<br>Unbinned pixel size	31,000x<br>1.21 Å/pixel
Camera	K2 Summit (Gatan Inc)
Exposure time	8s
Number of frames	40
Total dose ($e^-/Å^2$)	40.0
**Image processing of the capsid**	
EMDB	13120
Symmetry	I
Final number of Particles	3399
Map resolution in Å (FSC 0.143)	3.9
**Model statistics for the capsid**	
PDB	7OZ4
Model to map resolution in Å (FSC 0.5)	4.0
Ramachandran favored (%)	92.1
Ramachandran outliers (%)	0.0
Rama Z score	-1.87
Rotamer outliers (%)	0.0
C-beta deviations	0
Rms on bond lengths	0.0102
Rms on bond angles	1.02
Clashscore	6.28
Molprobity score	1.83
